# CaMKIIdelta subtypes: localization and function

**DOI:** 10.3389/fphar.2014.00015

**Published:** 2014-02-11

**Authors:** Charles B. B. Gray, Joan Heller Brown

**Affiliations:** ^1^Department of Pharmacology, University of California at San Diego, San DiegoCA, USA; ^2^Biomedical Sciences Graduate Program, University of California at SanDiego, SanDiegoCA, USA

**Keywords:** Ca^2+^/calmodulin-dependent protein kinase II, heart, splice variants, nuclear localization, transgenic mice

## Abstract

In this review we discuss the localization and function of the known subtypes of calcium/calmodulin dependent protein kinase IIδ (CaMKIIδ) and their role in cardiac physiology and pathophysiology. The CaMKII holoenzyme is comprised of multiple subunits that are encoded by four different genes called CaMKIIα, β, γ, and δ. While these four genes have a high degree of sequence homology, they are expressed in different tissues. CaMKIIα and β are expressed in neuronal tissue while γ and δ are present throughout the body, including in the heart. Both CaMKIIγ and δ are alternatively spliced in the heart to generate multiple subtypes. CaMKIIδ is the predominant cardiac isoform and is alternatively spliced in the heart to generate the CaMKIIδ_B_ subtype or the slightly less abundant δ_C_ subtype. The CaMKIIδ_B_ mRNA sequence contains a 33bp insert not present in δ_C_ that codes for an 11-amino acid nuclear localization sequence. This review focuses on the localization and function of the CaMKIIδ subtypes δ_B_ and δ_C_ and the role of these subtypes in arrhythmias, contractile dysfunction, gene transcription, and the regulation of Ca^2+^ handling.

## EXPRESSION AND LOCALIZATION

Calcium/calmodulin dependent protein kinase II (CaMKII) is a multimeric enzyme consisting of distinct subunits encoded by four different genes known as CaMKIIα, β, γ, and δ. These genes have a high degree of sequence homology but show differential tissue expression. CaMKIIα and β are predominantly expressed in neuronal tissue while γ and δ are present throughout the body, including the heart (Bennett et al., 1983; Tobimatsu and Fujisawa, 1989). CaMKIIδ is the predominant cardiac isoform and is alternatively spliced to generate multiple subtypes (Edman and Schulman, 1994).

**FIGURE 1:**
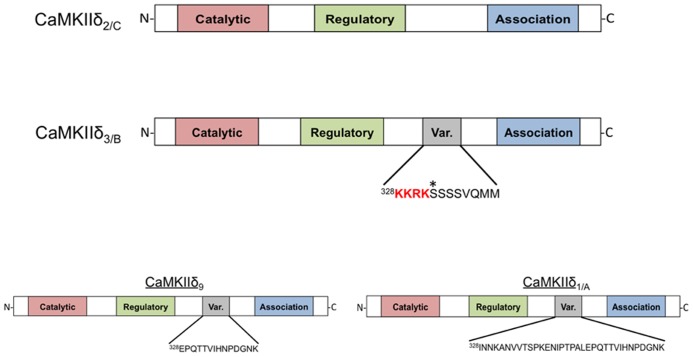
**Domain scheme of CaMKIIδ monomers.** The four subtypes shown are distinguished by the amino acid sequence of a variable region between the regulatory and association domains. CaMKIIδ_2/C_ lacks this variable domain while CaMKIIδ_3/B_ contains an 11-amino acid insertion that contains the canonical nuclear localization signal (NLS) sequence KKRK. An asterisk denotes a phosphorylation site (Ser^332^) in this domain that affects NLS accessibility. CaMKIIδ_9_ contains a different variable domain sequence that is also present in the longer variable region of CaMKIIδ_1/A_. All of these subtypes can be detected in adult murine heart with the exception of CaMKIIδ_1/A_, which is normally only present in neonatal mouse heart.

Schworer et al. (1993) were the first to demonstrate that there are different subtypes of CaMKIIδ expressed in various tissues. The authors reported four distinct proteins with differential expression patterns and named them CaMKIIδ_1-4_. CaMKIIδ_2_, and CaMKIIδ_3_ were shown to be identical except for the insertion of an 11-amino acid sequence in the variable domain of CaMKIIδ_3_, the more abundant of the two subtypes in the heart (Schworer et al., 1993). Around the same time, Edman and Schulman (1994) identified these same CaMKIIδ subtypes in rat heart and characterized their catalytic activity and regulation by calcium-liganded calmodulin (Ca^2+^/CaM). They refer to the predominant cardiac subtypes as CaMKIIδ_B_ and CaMKIIδ_C_ (the convention that will be used in this review), which correspond to the δ_3_ and δ_2_ subtypes, respectively. The structure of these proteins is shown in **Figure 1**. CaMKIIδ_B_ and δ_C_ possess similar catalytic activity and sensitivity to Ca^2+^/CaM. Furthermore, both subtypes can undergo autophosphorylation and acquire a similar degree of Ca^2+^-independent or autonomous activity (Edman and Schulman, 1994). In the years that followed, seven additional splice variants of the CaMKIIδ gene, referred to as CaMKIIδ_5-11_, were identified. Only one of these, CaMKIIδ_9_, is expressed in the adult heart (**Figure 1**; Mayer et al., 1994, 1995; Hoch et al., 1998, 1999).

The 11-amino acid insert in CaMKIIδ_B_ (^328^KKRKSSSSVQMM) is also present in some splice variants of CaMKIIα and γ; this conservation suggests an important function (Schworer et al., 1993). Srinivasan et al. (1994) showed that when constructs of CaMKIIδ_B_ are transfected into fibroblasts the expressed protein is localized to the nucleus. This is not the case for constructs of CaMKIIδ_C_, implying that that the additional amino acid sequence present in CaMKIIδ_B_ is responsible for nuclear localization (Srinivasan et al., 1994). A similar differential localization pattern was also observed when CaMKIIδ subtypes were expressed neonatal rat ventricular myocytes (NRVMs; Ramirez et al., 1997). Further studies showed that the 11-amino acid insert in CaMKIIδ_B_ can confer nuclear localization when inserted into the variable domain of CaMKIIα and that mutagenesis of the first two lysines in the insert abrogates the nuclear localization of these constructs. Thus it is widely accepted that the CaMKIIδ_B_ variable domain contains a nuclear localization signal (NLS).

CaMKII heteromultimerization is permissive in that the CaMKII holoenzyme can include subunits from multiple CaMKII genes and multiple splice variants of those genes (Bennett et al., 1983; Yamauchi et al., 1989). It seems likely that more than a single CaMKIIδ subtype is present in a single CaMKIIδ multimer and accordingly the ratio of δ_B_ to δ_C_ in a multimer could regulate the localization of the holoenzyme. This has been demonstrated experimentally. When CaMKIIδ_B_ and δ_C_ are cotransfected into fibroblasts or NRVMs, the localization of the expressed protein can be shifted in accordance with the ratio of the expressed CaMKIIδ subtypes, i.e., highly expressed δ_C_ sequesters δ_B_ in the cytosol and blocks its nuclear localization (Srinivasan et al., 1994; Ramirez et al., 1997). The opposite is also true: high relative expression of δ_B_ can localize δ_C_ to the nucleus. While not well appreciated, CaMKIIδ_B_ localization can also be regulated by phosphorylation. A serine (Ser^332^) immediately adjacent to the NLS of CaMKIIδ_B_ was shown to be a site of phosphorylation by CaMKI and CaMKIV *in vitro*. Phosphorylation prevents association of δ_B_ with the NLS receptor m-pendulin and thus limits localization of CaMKIIδ_B_ to the nucleus (Heist et al., 1998). Remarkably this mode of regulation is also seen when the NLS is moved from the middle of the protein to the N-terminus, suggesting that conformational changes are not required for phosphorylation to block the NLS.

Relative expression of CaMKIIδ subtypes is altered during cardiomyocyte differentiation and maturation and in association with the development of heart failure and ischemia/reperfusion (I/R) injury (Hoch et al., 1998, 1999; Colomer et al., 2003; Peng et al., 2010). In humans CaMKIIδ_B_ mRNA is selectively upregulated during heart failure (Hoch et al., 1999). The altered expression of particular subtypes suggests the possibility of a regulated process governing CaMKIIδ mRNA splicing because transcriptional regulation would not be expected to alter the ratio of CaMKIIδ subtypes. Alternative splicing factor/pre-mRNA-splicing factor SF2 (ASF/SF2) was initially described by Krainer and Maniatis (1985) and subsequently mice lacking ASF/SF2 expression were demonstrated to have incomplete processing of CaMKIIδ mRNA (Krainer and Maniatis, 1985; Xu et al., 2005). Specifically, enhanced expression of the δ_A_ subtype [δ_1_ in the nomenclature of Schworer et al. (1993)] was observed while expression of CaMKIIδ_B_ and δ_C_ was diminished. **Figure 1** also depicts the structure of the δ_A_ subtype, which is expressed in the fetal heart. ASF/SF2 can be regulated by phosphorylation. Protein kinase A (PKA)-mediated ASF/SF2 phosphorylation has been correlated with alternative splicing of CaMKIIδ in heart and brain (Gu et al., 2011). Additionally, regulation of ASF/SF2 by Protein phosphatase 1 γ (PP1γ) has been demonstrated to affect CaMKIIδ splicing (Huang et al., 2013). CaMKIIδ_A_ expression is increased in models of isoproterenol-induced cardiac hypertrophy and thus regulation of CaMKIIδ splicing by PKA and PP1γ may be relevant in the context of chronic β-adrenergic stimulation (Li et al., 2011). The RNA binding proteins Fox 1 (RBFOX1) and 2 (RBFOX2) collaborate with ASF/SF2 to induce proper CaMKIIδ splicing (Han et al., 2011) and factors that regulate these proteins could also influence the expression of CaMKIIδ subtypes. Thus, CaMKIIδ splicing is a dynamic and regulated process. The role of this system in the heart has not been extensively explored but could be of major importance since regulation of CaMKIIδ splicing may account for altered subtype expression and CaMKIIδ signaling in physiological and pathophysiological settings.

## CaMKIIδ_B_ TRANSGENIC MICE

The differential localization and function of CaMKIIδ subtypes could be of considerable importance to understanding the role of this enzyme in normal physiology and disease states. Early studies demonstrated that expression of CaMKIIδ_B_ in NRVMs induced atrial natriuretic factor (ANF) expression and led to increased myofilament organization, both hallmarks of cardiac hypertrophy, while expression of CaMKIIδ_C_ did not (Ramirez et al., 1997). This finding suggested that nuclear CaMKIIδ localization is required to regulate gene expression. Consistent with this notion are data indicating that CaMKIIδ_B_ signaling activates several transcription factors including myocyte enhancer factor 2 (MEF2), GATA4, and heat shock factor 1 (HSF1; Little et al., 2009; Lu et al., 2010; Peng et al., 2010). The significance of the hypertrophic responses elicited by δ_B_ *in vitro* was explored further by generation of CaMKIIδ_B_ transgenic (TG) mice (Zhang et al., 2002). These animals, which overexpress δ_B_ under the control of the cardiac-specific α-myosin heavy chain (α-MHC) promoter, demonstrate the enhanced expression of hypertrophic markers observed in NRVMs expressing CaMKIIδ_B_. CaMKIIδ_B_TG animals develop hypertrophy and moderate cardiac dysfunction by 4 months of age. Thus, CaMKIIδ_B_ expression appears to be sufficient to induce cardiac hypertrophy. Surprisingly, despite the increased CaMKII activity in the CaMKIIδ_B_TG mouse heart, phosphorylation of the canonical cardiac CaMKII substrate phospholamban (PLN) at its CaMKII site (Thr^17^) was not increased but rather was decreased relative to WT mice. PLN phosphorylation at the PKA site (Ser^16^) was similarly reduced. These data were related to increases in phosphatase activity (Zhang et al., 2002), but also implied that CaMKIIδ_B_ did not lead to robust phosphorylation of PLN. A subsequent paper that examined CaMKIIδ_B_TG animals at a younger age to avoid changes in phosphatase activity confirmed that phosphorylation of PLN and another cardiac CaMKII substrate, the cardiac ryanodine receptor (RyR2), was not increased by cardiac CaMKIIδ_B_ expression (Zhang et al., 2007). This finding is consistent with a predominantly nuclear localization and function of the δ_B_ subtype.

CaMKIIδ_B_ has also been suggested to regulate expression of the Na^+^/Ca^2+^ exchanger (NCX1) during the development of cardiac dysfunction following trans-aortic constriction (TAC; Lu et al., 2011). The conclusion that δ_B_ was the subtype involved in NCX1 regulation relied on the use of a constitutively active construct of CaMKIIδ_B_ in which a Thr to Asp mutation (T287D) simulates autophosphorylation. Interestingly, the authors found that this construct was excluded from the nucleus (Lu et al., 2010). This differs from the localization pattern described above (Srinivasan et al., 1994; Ramirez et al., 1997) and can be explained as a result of phosphorylation of Ser^332^ in the 11-amino acid insert of δ_B_ (**Figure 1**). The observation that mutation of Ser^332^ to Ala restores nuclear localization of constitutively active CaMKIIδ_B_ (Backs et al., 2006) confirms the role of this site in the cytosolic localization of the active construct. The possibility that phosphorylation of Ser^332^ might regulate CaMKIIδ_B_ localization in the intact heart has not been evaluated, but such a mechanism could contribute to the observation that CaMKIIδ_B_ is found outside the nucleus even in the absence of multimerization with δ_C_**(Mishra et al., 2011).

## CaMKIIδ_C_ TRANSGENIC MICE

CaMKIIδ_C_ transgenic mice have also been generated and demonstrate a strikingly different phenotype from mice that express CaMKIIδ_B_. While cardiac dysfunction is relatively moderate and takes months to develop in CaMKIIδ_B_TG animals, mice expressing δ_C_ rapidly progress to heart failure and premature death (Zhang et al., 2003). By 6 weeks of age CaMKIIδ_C_TG animals display marked changes in cardiac morphology and by 12 weeks these animals display severe cardiac dysfunction and upregulation of hypertrophic genes.****

### Ca^2+^ HANDING AND ARRHYTHMIA

**FIGURE 2:**
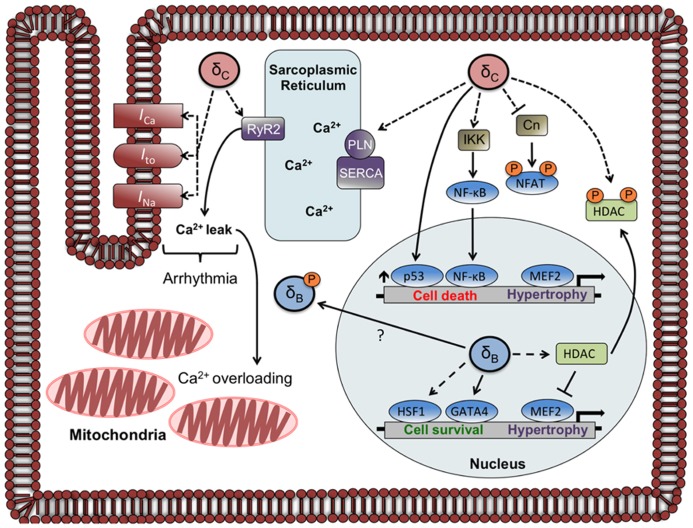
**Localization and function of CaMKIIδ subtypes in the adult cardiomyocyte.** The circles labeled δ_C_ and δ_B_ represent CaMKIIδ multimers that are composed primarily of δ_C_ and δ_B_ subunits, respectively. Documented phosphorylation events are indicated by dashed lines. CaMKIIδ_C_ regulates Ca^2+^ homeostasis and currents involved in arrhythmogenesis through phosphorylation of Ca^2+^ handling proteins and channels. CaMKIIδ_C_ can also affect gene transcription through direct and indirect mechanisms including phosphorylation of NFAT and HDAC (sequestering them in the cytosol), increases in p53, and increased nuclear import of NF-κB. The CaMKIIδ_B_ subtype has little effect on phosphorylation of Ca^2+^ handling proteins but increases gene expression through HDAC phosphorylation and nuclear export and activation of HSF1 and GATA4. A putative mechanism for δ_B_redistribution is depicted, showing δ_B_exiting or being excluded from the nucleus due to phosphorylation at a site (Ser^332^) adjacent to its NLS.

Expression of the cardiac sarco/endoplasmic reticulum Ca^2+^-ATPase (SERCA) is diminished in δ_C_TG mice as occurs in other models of heart failure. Since SERCA regulates Ca^2+^ reuptake into the sarcoplasmic reticulum (SR), this decrease would diminish SR Ca^2+^ loading. On the other hand, the CaMKIIδ_C_TG mice show hyperphosphorylation of PLN at Thr^17^, which should improve SERCA function. In addition δ_C_TG animals display marked increases in phosphorylation of the RyR2, the channel through which Ca^2+^ exits the SR. Taken together, these changes would predict dysregulation of SR Ca^2+^ cycling and excitation–contraction coupling. This was substantiated in an accompanying paper that systematically analyzed and demonstrated dysregulation of cardiac Ca^2+^ handling in mice expressing δ_C_ (Maier et al., 2003). Specifically it was shown that SR Ca^2+^ stores were depleted in myocytes from these animals, explaining the observation that isolated myocytes displayed diminished twitch shortening amplitude. Furthermore, Maier et al. (2003) showed that the frequency and duration of Ca^2+^ sparks, or spontaneous intracellular Ca^2+^-release events, was markedly increased in myocytes from animals expressing δ_C_. Hyperphosphorylation of RyR2 by CaMKIIδ_C_ was hypothesized to underly the enhanced leak of Ca^2+^ from the SR, and this was verified by the demonstration that acute inhibition of CaMKII in δ_C_TG myocytes rescues the altered Ca^2+^ handling (Maier et al., 2003). In other experiments, acute expression of δ_C_ in rabbit cardiomyocytes was shown to be sufficient to induce SR Ca^2+^ sparks and diminished SR Ca^2+^ loading (Kohlhaas et al., 2006). These findings imply that direct regulation of Ca^2+^ handling targets including RyR2 by CaMKIIδ_C_ can account for the dysregulation of Ca^2+^ and contractile function seen in myocytes from δ_C_TG animals (**Figure 2**).

Dysregulation of excitation–contraction coupling by CaMKII is thought to contribute to arrythmogenesis in a variety of contexts, as supported by the increased incidence of arrhythmogenic events in CaMKIIδ_C_TG mice (Anderson et al., 1998; Wu et al., 2002; Wagner et al., 2006). Overexpression of CaMKIIδ_C_ not only induces more spontaneous arrhythmias but also enhances the susceptibility of mice to arrhythmogenic challenge by β-adrenergic stimulation. Sag et al. (2009) found that much of the proarrhythmogenic effects of β-adrenergic stimulation on SR Ca^2+^ leak were significantly inhibited by treatment of myocytes with KN-93, an inhibitor of CaMKII. Furthermore the SR Ca^2+^ leak induced by isoproterenol did not occur in myocytes from mice lacking CaMKIIδ. These findings collectively implicate SR Ca^2+^ leak as one of the key mechanisms in δ_C_-mediated arrhythmias (Sag et al., 2009). The notion that hyperphosphorylation of RyR2 at the CaMKII site (Ser^2814^) contributes to arrhythmias and SR Ca^2+^ leak is supported by the finding that mutation of Ser^2814^ to Ala (S2814A) blocks the ability of CaMKII to induce Ca^2+^ sparks (van Oort et al., 2010). The autosomal dominant form of catecholaminergic polymorphic ventricular tachycardia (CPVT) can be caused by the RyR2 mutation R4496C and mice carrying this mutation are predisposed to arrhythmia and ventricular fibrillation. Enhanced CaMKIIδ_C_ expression and activity are implicated in the etiology of premature death in CPVT as expression of CaMKIIδ_C_ exacerbates the effects of the R4496C mutation (Dybkova et al., 2011). As mentioned earlier RyR2 Ser^2814^ phosphorylation is increased by expression of CaMKIIδ_C_ (but not by δ_B_) *in vivo* (Zhang et al., 2007) and the effects of mutating this site (van Oort et al., 2010) emphasize the importance of RyR2 phosphorylation by CaMKII in SR Ca^2+^ leak and arrhythmia.

Other targets besides those at the SR may contribute to the arrhythmogenic phenotype of CaMKIIδ_C_ mice. The cardiac sodium channel Na_V_1.5 is physically associated with CaMKIIδ_C_ based on coimmunoprecipitation of these proteins from CaMKIIδ_C_TG animals and Na_V_1.5 is phosphorylated in mice expressing δ_C_ (Wagner et al., 2006). CaMKIIδ_C_ phosphorylates Na_V_1.5 at multiple sites and phosphorylation appears to elicit the loss-of-function changes in Na_V_1.5 gating that are observed in the context of CaMKIIδ_C_ expression *in vitro *(Ashpole et al., 2012; Koval et al., 2012). Incomplete inactivation of Na_V_1.5 generates a late Na^+^ current (*I*_Na_), which can prolong the duration of the action potential and contribute to arrhythmias. Additionally, increased *I*_Na_ can lead to Na^+^-overloading of the cardiomyocyte, which contributes to diminished diastolic contractile performance (Maltsev et al., 1998). Late *I*_Na_ is observed in CaMKIIδ_C_TG mice and inhibition of this current ameliorates arrhythmia and diastolic dysfunction in these animals (Sossalla et al., 2011). Modulation of *I*_Na_ therefore appears to contribute to the phenotype of CaMKIIδ_C_ mice with respect to arrhythmia development; additionally the CaMKIIδ_C_ subtype likely regulates the L-type Ca^2+^ channel (LTCC) and repolarizing potassium currents (*I*_to_ and *I*_K1_; McCarron et al., 1992; Wagner et al., 2009). Thus, a multitude of mechanisms link CaMKIIδ_C_ to arrhythmogenesis.

### CONTRACTILE DYSFUNCTION

Arrhythmias may contribute to the premature death of CaMKIIδ_C_TG mice but there are also marked decreases in contractile function in these animals. Since alterations to cardiomyocyte Ca^2+^ handling are seen in relatively young CaMKIIδ_C_TG mice and precede the development of heart failure, it is possible that dysregulated Ca^2+^ homeostasis (specifically SR Ca^2+^ leak) is an initiating event in δ_C_-induced heart failure. Specifically, as a consequence of SR Ca^2+^ leak and SERCA downregulation, the SR Ca^2+^ load is diminished which would compromise contractile function. To determine whether diminished SR Ca^2+^ load is the primary causal event leading to contractile dysfunction and premature death in response to δ_C_ overexpression, we crossed the δ_C_TG mice with mice in which the SERCA regulatory protein PLN was deleted (PLN-KO). Deletion of PLN in the context of δ_C_ overexpression normalized SR Ca^2+^ levels and the contractile function of isolated myocytes was restored (Zhang et al., 2010). Remarkably the development of cardiac dysfunction *in vivo* was not rescued but instead was accelerated in the δ_C_TG/PLN-KO mice. In addition SR Ca^2+^ leak was enhanced. It was hypothesized that the increased SR Ca^2+^ load, in the context of RyR2 hyperphosphorylation, precipitated greater Ca^2+^ leak and further suggested that the accelerated development of cardiac dysfunction was due to mitochondrial Ca^2+^ overloading (Zhang et al., 2010). These observations and their interpretation places central importance on the Ca^2+^ leak elicited by δ_C_-mediated phosphorylation of RyR2 in the development of heart failure. Further support for this hypothesis comes from the finding that CaMKIIδ knockout mice have attenuated contractile dysfunction in response to pressure overload induced by TAC and myocytes from these animals show diminished SR Ca^2+^ leak in response to TAC (Ling et al., 2009). Additionally, mice expressing the RyR2 S2814A mutation are protected from the development of heart failure in response to pressure overload (Respress et al., 2012) consistent with a critical role for CaMKII-mediated RyR2 phosphorylation. We recently crossed CaMKIIδ_C_ mice with those expressing RyR2 S2814A; if the hypothesis is correct these mice will show diminished SR Ca^2+^ leak and improved contractile function when compared to CaMKIIδ_C_TG mice.

Another approach used to determine the role of RyR2 phosphorylation and SR Ca^2+^ leak in the phenotype of CaMKIIδ_C_TG mice was to cross the CaMKIIδ_C_TG mice with mice expressing SR-targeted autocamtide-2-related inhibitory peptide (SR-AIP; Huke et al., 2011). AIP simulates the regulatory domain of CaMKII and inhibits the kinase, and SR-AIP mice have been shown to display diminished phosphorylation of CaMKII substrates at the SR (Ji et al., 2003). A reduction in the extent of PLN and RyR2 hyperphosphorylation observed in CaMKIIδ_C_TG mice was conferred by SR-AIP. There were associated changes in Ca^2+^ handling that indicated a modest improvement in SR Ca^2+^ leak. Despite the salutary effects of SR-AIP in cells from δ_C_TG mice, *in vivo* cardiac function was not improved. One possible explanation for these findings is that the degree of inhibition of RyR2 phosphorylation conferred by SR-AIP was insufficient to prevent the effects of CaMKIIδ_C_ overexpression. Alternatively, while δ_C_-mediated phosphorylation of targets at the SR including RyR2 and PLN is of considerable consequence, targets of CaMKII elsewhere in the cell may also contribute to the pathogenesis of cardiac dysfunction induced by CaMKIIδ_C_.

Mitochondrial Ca^2+^ is elevated in mice overexpressing δ_C_ in the context of intact SR Ca^2+^ load (Zhang et al., 2010) and increases in mitochondrial Ca^2+^ are known to induce opening of the mitochondrial permeability transition pore (MPTP) and cell death (Halestrap and Davidson, 1990). Considering the central importance of mitochondria in the regulation of cell death and of cell death in the development of heart failure (Wencker et al., 2003), any pathway by which CaMKIIδ_C_ induces mitochondrial Ca^2+^ overloading and subsequent loss of mitochondrial integrity would be predicted to contribute to the development of contractile dysfunction and heart failure. To test the role of mitochondrial dysregulation in the cardiomyopathy that develops in δ_C_TG animals, CaMKIIδ_C_TG mice were crossed with mice lacking expression of cyclophilin D, a mitochondrial protein required for the formation of the MPTP. The ability of high Ca^2+^ to induce swelling of isolated mitochondria, an index of MPTP opening, was impaired in the CaMKIIδ_C_TG mice lacking cyclophilin D, but development of dilated cardiomyopathy and premature death of these mice was not diminished. Indeed these responses were exacerbated when compared to δ_C_TG mice with intact cyclophilin D expression. The authors suggest that cyclophilin D may actually play a beneficial role in stress responses, as they observed that TAC-induced heart failure development was also made more severe by genetic deletion of cyclophilin D (Elrod et al., 2010). However, CaMKIIδ_C_ is found at mitochondria and a recent seminal study by Joiner et al. (2012) identified the mitochondrial Ca^2+^ uniporter (MCU) as a potential target of CaMKII (Mishra et al., 2011; Joiner et al., 2012). While phosphorylation of the MCU by CaMKII was not shown to occur *in vivo*, a CaMKII-dependent change in the function of the MCU was evidenced by data demonstrating that a CaMKII inhibitory peptide targeted to the mitochondria diminished mitochondrial Ca^2+^ uptake and inhibited apoptosis in mice subjected to myocardial infarction and I/R injury.

## CaMKIIδ SUBTYPES IN GENE TRANSCRIPTION

The discussion above, and indeed most of the literature, considers the role of CaMKIIδ-mediated phosphorylation and regulation of Ca^2+^ handling proteins and ion channels. Chronic elevations in CaMKIIδ expression and activity are observed in humans with heart failure (Hoch et al., 1999) and these long-term changes are likely to elicit altered gene expression. As discussed earlier, CaMKIIδ_B_ induces the expression of hypertrophic genes in myocytes and transgenic mice, consistent with its primarily nuclear localization (Ramirez et al., 1997; Zhang et al., 2002). Other work showed that the CaMKIIδ_B_ subtype is required for GATA-4 binding to the B cell lymphoma 2 (Bcl-2) promoter and subsequent gene expression (Little et al., 2009). Furthermore, CaMKIIδ_B_ was shown to phosphorylate the transcription factor HSF1 thereby increasing its transcriptional activity (Peng et al., 2010). Taken together, these observations imply that it is the CaMKIIδ_B_ subtype that regulates gene expression as a result of its actions in the nucleus.

It is not necessarily the case, however, that gene regulation requires CaMKIIδ to be localized to the nuclear compartment. Despite its primarily cytosolic localization, CaMKIIδ_C_ overexpressed in mouse heart increased phosphorylation of histone deacetylase 4 (HDAC4), resulting in activation of the transcription factor MEF2 (Zhang et al., 2007). CaMKIIδ_C_ has also been demonstrated to regulate nuclear localization of nuclear factor of activated T cells (NFATs) in NRVM. The ability of CaMKIIδ_C_ to decrease nuclear NFAT was blocked by coexpression of a dominant-negative construct of CaMKIIδ_C_ and was shown to be elicited by phosphorylation and inhibition of the Ca^2+^/CaM dependent phosphatase calcineurin (Cn; MacDonnell et al., 2009), presumably in the cytosol. Alteration of Ca^2+^ homeostasis by cytosolic CaMKIIδ_C_ expression may indirectly affect gene expression and additionally the constitutively active CaMKIIδ_B_ utilized in the studies discussed above (Lu et al., 2011) is cytosolic and yet regulates expression of NCX1.

Regulation of gene expression by CaMKIIδ_B_ has been demonstrated to promote cardiomyocyte survival while the opposite is true for CaMKIIδ_C_. CaMKIIδ_B_ was shown to protect cardiomyocytes from doxorubicin-induced cell death via transcriptional upregulation of Bcl-2 (Little et al., 2009). Along similar lines, CaMKIIδ_B_ contributes to cardioprotection from H_2_O_2_ by increasing inducible heat shock protein 70 (iHSP70) expression (Peng et al., 2010). Conversely, CaMKIIδ_C_ activation is implicated in cell death elicited by a variety of stimuli (Zhu et al., 2007). It has been suggested that CaMKIIδ_C_ (but not δ_B_) upregulates the proapoptotic transcription factor p53 (Toko et al., 2010), and recent work from our laboratory demonstrates that CaMKIIδ_C_ expression in NRVMs activates the proinflammatory transcription factor nuclear factor κB (NF-κB; Ling et al., 2013). We demonstrated that CaMKIIδ_C_ increased phosphorylation of IκB Kinase (IKK) and since IKK activation can also upregulate p53(Jia et al., 2013), this pathway may contribute to the proapoptotic response reported by Toko et al. (2010).

## FUTURE DIRECTIONS

There is compelling evidence that the CaMKIIδ_B_ and δ_C_ subtypes differentially regulate cardiomyocyte Ca^2+^ handling and survival *in vitro*. Whether this occurs *in vivo* under physiological or pathophysiological conditions, and whether δ_B_ and δ_C_ subserve different functions based on their localization or selective activation, remains to be determined.

It seems likely that the relative levels of endogenous δ_B_ and δ_C_ determine localization and could therefore impact CaMKIIδ signaling. Hypothetically, a selective increase in CaMKIIδ_C_ would result in accumulation of cytosolic CaMKIIδ and depletion of nuclear CaMKIIδ while a selective increase in CaMKIIδ_B_ would have the opposite effect. CaMKIIδ redistribution in this manner may contribute to the phenotype of mice that overexpress δ_B_ and δ_C_ and importantly there are changes in the relative expression of δ_B_ and δ_C_ in models of heart failure and I/R injury. In both models δ_C_ expression is enhanced relative to that of δ_B_ (Zhang et al., 2003; Peng et al., 2010). It is not known how this occurs but it is of interest to postulate that in heart failure and during I/R regulation of CaMKIIδ splicing is altered. ASF/SF2 and RBFOX1/2 regulate the splicing of the CaMKIIδ gene and thus expression of δ_B_ and δ_C_, but whether changes in splicing occur in and contribute to the development of heart failure or I/R injury remains to be determined. It is likely that the increased δ_C_ expression observed in these models is pathogenic.

While CaMKIIδ_B_ contains an NLS, this subtype is not completely sequestered in the nucleus (Mishra et al., 2011). As mentioned previously the NLS within the variable domain of δ_B_ can be regulated by phosphorylation, which prevents nuclear localization. This type of regulation could be of considerable importance since the nuclear localization of δ_B_ appears to correlate with enhanced expression of protective genes and cell survival while cytosolic localization does not (Little et al., 2009; Peng et al., 2010; Lu et al., 2011).

Of additional interest is the neglected CaMKIIδ_9_. The pioneering work of (Hoch et al., 1998; Mayer et al., 1995) identified δ_9_ as one of the three subtypes of CaMKIIδ in the adult heart and showed that it is expressed at similar levels to those of CaMKIIδ_B_. δ_9_ contains a sequence (^328^EPQTTVIHNPDGNK) not present in δ_B_ or δ_C_ and thus may possess unique properties that merit further investigation, as the function and localization of δ_9_ *in vivo* has not been explored. Along similar lines, CaMKIIδ_A_ expression is increased in a model of cardiac hypertrophy (Li et al., 2011), but the possibility that this splice variant is upregulated in and contributes to cardiovascular disease has not been investigated.

## Conflict of Interest Statement

The authors declare that the research was conducted in the absence of any commercial or financial relationships that could be construed as a potential conflict of interest.
